# Stable Coronary Artery Disease: Who Finally Benefits from Coronary Revascularization in the Modern Era? The ISCHEMIA and Interim ISCHEMIA-EXTEND Analysis

**DOI:** 10.3390/life13071497

**Published:** 2023-07-01

**Authors:** Leonid Bershtein, Alexey Sumin, Elizaveta Zbyshevskaya, Victoria Gumerova, Darejan Tsurtsumia, Igor Kochanov, Alina Andreeva, Vartan Piltakian, Sergey Sayganov

**Affiliations:** 1 Department of Internal Medicine & Cardiology, North-Western State Medical University named after II Mechnikov, 191015 St. Petersburg, Russia; leonid.bershtein@szgmu.ru (L.B.); e.zbyshevskaya@szgmu.ru (E.Z.); vvoron1@yahoo.com (V.G.); 2 Federal State Budgetary Institution “Research Institute for Complex Issues of Cardiovascular Disease”, Sosnovy Blvd. 6, 650002 Kemerovo, Russia; 3 Department of Internal Medicine #1, North-Western State Medical University named after II Mechnikov, 191015 St. Petersburg, Russia; d.tcurtcumiya@szgmu.ru; 4 Department of Interventional Cardiology, North-Western State Medical University named after II Mechnikov, 191015 St. Petersburg, Russia; igor.kochanov@szgmu.ru; 5 Department of Functional Diagnostics, North-Western State Medical University named after II Mechnikov, 191015 St. Petersburg, Russia; doc.alina@mail.ru; 6 St. Petersburg State Budgetary Healthcare Institution ‘Pokrovskaya City Hospital’, 199034 St. Petersburg, Russia; vaite1984@icloud.com; 7 North-Western State Medical University named after II Mechnikov, 191015 St. Petersburg, Russia; sergey.sayganov@szgmu.ru

**Keywords:** chronic coronary syndromes, revascularization, prognosis, ISCHEMIA, ISCHEMIA-EXTEND

## Abstract

Coronary revascularization is one of the most studied treatment modalities in cardiology; however, there is no consensus among experts about its indications in patients with stable coronary artery disease (SCAD). Contemporary data regarding the role of revascularization in SCAD are in clear conflict with the current European guidelines. This article discusses the main statements of the most significant American and European Guidelines on myocardial revascularization of the last decade and also analyzes the appropriateness of revascularization to improve the prognosis and symptoms in SCAD in the light of new research data, primarily the ISCHEMIA study (NCT01471522) and the ACC/AHA 2021 Revascularization Guidelines based on them. Data on the revascularization in SCAD obtained after the completion of ISCHEMIA (including the interim analysis of ISCHEMIA-EXTEND) and their potential significance are discussed. The results of ISCHEMIA sub-analyses in the most important “controversial” subgroups (3-vessel disease, proximal left anterior descending artery disease, strongly positive stress test, etc.) are reviewed, as are the results of the ISCHEMIA-CKD substudy in patients with severe chronic kidney disease (CKD).

## 1. Introduction

A record number of studies have been conducted on myocardial revascularization in SCAD [1], but despite this, the choice between a conservative and invasive strategy in a particular patient remains a complex and controversial issue. The current European Society of Cardiology (ESC) Guidelines, containing sections on myocardial revascularization in SCAD (chronic coronary syndromes [CCS], in ESC terminology), published with an interval of one year [2,3], differ significantly. The recently completed large-scale randomized clinical trial (RCT) ISCHEMIA and the ongoing ISCHEMIA-EXTEND trial provide new evidence for treatment selection in SCAD that conflicts with the current guidelines. Moreover, the published results of ISCHEMIA have already become the subject of a new hot discussion arguing their role as the ultimate guide for decision-making [4,5]. Special attention is directed to the value of revascularization in subpopulations of known high risk, such as 3-vessel disease and unprotected left main coronary artery (LMCA) disease patients (the latter not presented in ISCHEMA) [6].

In this article, we analyze the appropriateness of myocardial revascularization to improve prognosis and symptoms in SCAD and compare the statements of the current Guidelines with the new research data.

## 2. Summary of Guidelines on Myocardial Revascularization before ISCHEMIA

The most significant recommendations on which clinical decision-making in practice has been based in the last decade were the European Guidelines on Myocardial Revascularization [2], the ESC Guidelines for the Diagnosis and Management of Chronic Coronary Syndromes [3], and the American Guideline for the Diagnosis and Management of Patients with Stable Ischemic Heart Disease [7]. Revascularization is performed to alleviate the ischemic symptoms or to improve the prognosis.

The indications for revascularization available in the listed sources are summarized below (Table 1 and text).

European revascularization Guidelines [2] are very similar. There, the Class I and IIa indications for myocardial revascularization to improve prognosis are high-risk coronary lesions: left main coronary artery (LMCA) disease; proximal stenosis of the left anterior descending artery (LAD); two or three vessel disease in patients with significantly depressed left ventricular ejection fraction (EF) of <35%; stenosis of the last patent coronary artery (all lesions are considered angiographically significant if ≥50% according to the European standard). Also, inducible ischemia of a large area (>10% of the left ventricle [LV] area) during a stress test is an indication for coronary intervention. To improve symptoms, revascularization is recommended in the presence of hemodynamically significant coronary artery stenosis if the patient has limiting angina or the angina equivalent (dyspnea) with an insufficient response to OMT while considering the patient’s preference. Revascularization of stenosis is warranted if it is >90% by angiography or if 50–90%, when (1) ischemia of >10% LV area is induced in this coronary territory at an imaging stress test or (2) measurement of FFR/instantaneous wave-free ratio (iwFR) gives a result of ≤0.80 or ≤0.89, respectively.

However, the ESC Guidelines on CCS [3] state in contrast that an invasive strategy may improve prognosis in any hemodynamically significant lesion. According to this document, revascularization may be considered in a patient who is asymptomatic (i.e., for prognostic purposes) when (1) ischemia is confirmed by a stress test and the area of inducible ischemia is >10% of the LV, or (2) when the stress test is negative or not carried out, in the presence of stenosis of any localization if hemodinamically significant (defined as above); revascularization should also be considered with an EF < 35% as a result of coronary artery disease. Also, revascularization may be indicated to improve symptoms in a patient with angina and confirmed myocardial ischemia.

Although these guidelines have been the basis of clinical decision-making for a long time, they are controversial. Also, statements on the prognostic benefit of coronary artery bypass grafting (CABG) in individuals with high-risk coronary lesions and reduced EF are based on observational data from the 1980s and 1990s, conducted before the era of modern optimal medical therapy (OMT) [8]. The prognostic advantage of percutaneous coronary intervention (PCI) over OMT was, in fact, only shown in the FAME 2 study, where revascularization was performed after confirming the hemodynamic significance of stenosis by measuring the FFR [9]. These data are the basis of the latest version of the European recommendations [3]. Nevertheless, FAME 2 failed to show differences in the “hard” endpoints of cardiac death, acute myocardial infarction (AMI), and confirmed unstable angina. The only parameter in which the invasive strategy showed superiority was acute coronary syndrome, not confirmed by ECG/biomarkers. The explanation for this is the increased alertness of patients in the “conservative” group of the FAME2 study (and their attending physicians) regarding any suspicious symptoms in a situation where the presence of significant stenosis was proven by FFR and revascularization, which in this case ‘should’ be performed, was not performed due to the conditions of the trial [10]. On the other hand, in the extended (5-year) follow-up of the FAME 2 cohort [11], the authors did show the advantage of PCI in terms of less urgent revascularizations and spontaneous AMIs. Obviously, if PCI of significant stenosis really prevents AMI, this conflicts with the existing idea of the predominant development of atherothrombosis at the site of an insignificant “vulnerable” atherosclerotic plaque [12].

## 3. Myocardial Revascularization: Evidence from the ISCHEMIA Trial

### 3.1. Myocardial Revascularization in SCAD and Prognosis

The ISCHEMIA study (NCT01471522) was the largest RCT (*n* = 5179) comparing an invasive and conservative strategy in patients with SCAD receiving contemporary medical therapy and PCI/CABG performed by contemporary approaches [13,14]. The study enrolled patients with a positive stress test of moderate or high risk. In 75% of cases, this was an imaging stress test. At the stress test, the mean peak exercise capacity was 7.8 METs, with a peak heart rate of 142 beats/min and a peak systolic blood pressure of 170 mmHg [15]. Coronary computed tomography angiography (CCTA) was performed to confirm the presence of obstructive (>50%) coronary artery disease, with LMCA involvement being the exclusion criterion. CCTA data were analyzed by the central laboratory and were not disclosed to the attending physician. Other major exclusion criteria were: absence of significant coronary artery stenosis; coronary anatomy not allowing PCI or CABG; EF < 35% (the mean EF of the enrolled patients was about 60%). Patients were randomized into groups of conservative or invasive therapy, with the last undergoing invasive coronary angiography and revascularization. The primary endpoint was a composite of death from cardiovascular causes, acute myocardial infarction (AMI), hospitalization for unstable angina, heart failure, or resuscitated cardiac arrest.

The type of revascularization was determined according to standard criteria, taking into account the SYNTAX score, surgical risk, comorbidity, and the physician’s and patient’s preferences [1,3]. Despite favorable RCT conditions, at the end of 3+ years of follow-up, only 41% of patients received true OMT (achievement of low-density lipoprotein cholesterol [LDL cholesterol] < 1.8 mmol/L, treatment with any statin, systolic blood pressure < 140 mm Hg, treatment with an antithrombotic drug, and no smoking), and the proportion of participants with a target level of LDL-C was 59%. As a method of revascularization, PCI using drug-eluting stents was performed in 74% of participants and CABG—using predominantly mammary grafts—in 26% of participants. The crossover rate from the conservative arm to the invasive arm was 28%, with 21% undergoing revascularization compared with 79% in the invasive arm [16].

The 7-item version of the Seattle Angina Questionnaire (SAQ) was used to assess symptoms [17,18].

**Figure 1:**
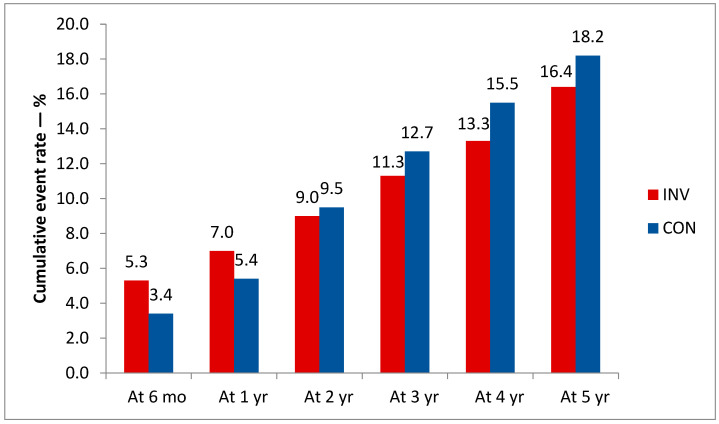
Cumulative primary endpoint event rate by treatment strategy and follow-up time. Estimated differences in event rates between the invasive and conservative groups at the indicated time points are: 1.9% (0.8 to 3.0); 1.5% (0.2 to 2.9); −0.5% (−2.1 to 1.1); −1.3% (−3.2 to 0.6); −2.2% (−4.4 to 0); −1.8% (−4.7 to 1.0). The 95% confidence intervals are shown in brackets.

The most important result of the ISCHEMIA study was the absence of a statistically significant difference between the invasive and conservative strategies in the cumulative incidence of cardiovascular events: adjusted hazard ratio (HR) for the invasive strategy: 0.93 (95% CI 0.80 to 1.08; *p* = 0.34). It should be noted that 2 years after the start of the follow-up, the event curves intersected, so that at the end of the observation there were more events in the conservative strategy group (Figure 1) [14,17].

The incidence of death from all causes in both groups was low: 145 (5.6%) in the invasive group and 144 (5.6%) in the conservative group (adjusted HR 1.05; 95% CI 0.83 to 1.32; *p* = 0.67). There wers more periprocedural AMIs in the invasive group (adjusted HR 2.98; 95% CI 1.87 to 4.74; *p* < 0.01), while there were less spontaneous AMIs by 33% (adjusted HR 0.67, 95% CI 0.53 to 0.87, *p* < 0.01).

**Figure 2:**
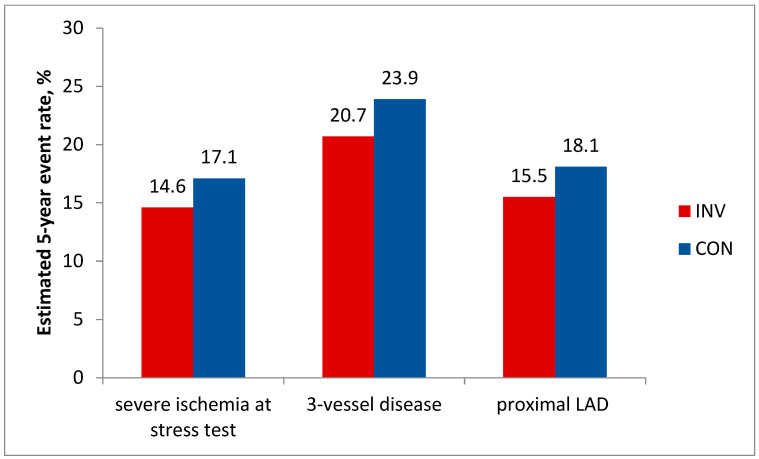
ISCHEMIA, analysis in prespecified important subgroups: Estimated 5-year cumulative primary endpoint rate, % by treatment strategy. *p* > 0.05 for all comparisons.

In addition, analyses in prespecified important subgroups showed no benefit of an invasive strategy regarding the primary endpoint in patients with severe stress-induced ischemia, 3-vessel disease, or proximal LAD disease (Figure 2).

The differences in the incidence of events between the invasive and conservative groups for these subgroups of patients were, respectively, −2.6% (95% CI −6.3% to1.2%); −3.2% (95% CI −9.5% to 3.2%); −2.6% (95% CI −7.5% to 2.3%); *p* > 0.05 for all.

### 3.2. Analysis of the Severity of Ischemia during a Stress Test and the Severity of Coronary Disease as Predictors of the Prognostic Benefit of an Invasive Strategy

To further explore the effect of ischemia severity and CAD severity on the primary endpoint, separate post hoc analyses were performed. The first one showed that the 4-year rates of all-cause mortality, spontaneous AMI, or the trial primary composite end point (cardiovascular death, hospitalization for heart failure, or hospitalization for unstable angina) were not statistically different between stress-induced ischemia subgroups [19]. For example, in patients with severe ischemia at the stress test, all-cause mortality was 6.1% (95% CI, 4.7% to 7.8%) in the invasive group vs. 5.6% (95% CI, 4.2% to 7.1%) in the conservative group; respectively, cardiovascular death was 3.7% (95% CI, 2.6% to 5.0%) vs. 4.5% (95% CI, 3.3% to 6.0%); spontaneous AMI occurred in 5.7% (95% CI, 4.4% to 7.3%) vs. 8.3% (95% CI, 6.8% to 10.0%); all *p* values for interaction were non-significant.

In the second analysis of the 4-year cumulative event rates by CAD severity and treatment assignment (in which only 48% of randomized participants could be included because CCTA was not carried out in low glomerular filtration rate (eGFR) and image quality was insufficient in some participants), the CAD-by-treatment strategy interaction *p* value for 4-year event rates was also >0.05 [19]. In the subgroup of participants with a modified Duke Prognostic Index score of 6 (i.e., 3-vessel severe stenosis (≥70%) or 2-vessel severe stenosis with proximal LAD), the 4-year estimated rate of all-cause mortality in the invasive and conservative groups was equal at 7.7%. In the same subgroup, the 4-year estimated rate of cardiovascular death was 3.7% (95% CI, 1.9 to 6.4%) in the invasive group vs. 6.7% (95% CI, 3.9 to 10.5%) in the conservative group. Finally, the 4-year estimated rate of spontaneous AMI was 5.4% (95% CI, 3.1 to 8.6%) vs. 10.2% (7.0 to 14.0%), respectively; all *p* values for interaction were non-significant.

## 4. ISCHEMIA-CKD

**Figure 3:**
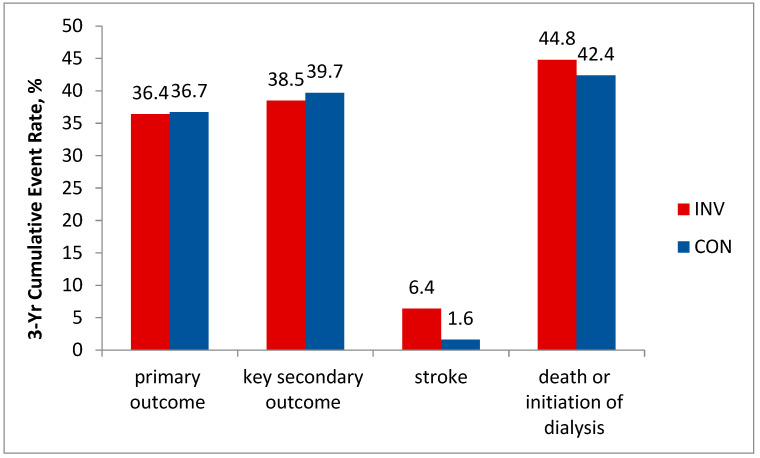
3-Yr Cumulative Event Rate in ISCHEMIA-CKD. *p* = NS for primary and key secondary outcomes. *p* = 0.004 for stroke; *p* = 0.03 for death or initiation of dialysis.

Severe CKD is usually an exclusion criterion in studies of patients with SCAD. Meanwhile, cardiovascular disease is the leading cause of death in patients with CKD [20]. On the other hand, while about 30% to 40% of all patients undergoing PCI have concomitant CKD, data on the optimal treatment strategies in this population remain scarce [21]. The ISCHEMIA-CKD substudy enrolled patients with advanced kidney disease (defined as an estimated eGFR of <30 mL per minute per 1.73 m^2^ of body surface area or being on dialysis) otherwise qualifying for the ISCHEMIA selection criteria [22]. According to the ISCHEMIA-CKD results, at a median follow-up of 2.2 years, an estimated 3-year event rate of the primary outcome event rate was 36.4% in the invasive group vs. 36.7% in the conservative group; adjusted HR, 1.01; 95% CI, 0.79 to 1.29; *p* = 0.95. Results for the key secondary outcome (composite of death, nonfatal AMI, hospitalization for unstable angina, heart failure, or resuscitated cardiac arrest) were also not in favor of the invasive strategy: 38.5% vs. 39.7%; adjusted HR, 1.01; 95% CI, 0.79 to 1.29. Moreover, the patients in the invasive group demonstrated a higher incidence of stroke (adjusted HR, 3.76; 95% CI, 1.52 to 9.32; *p* = 0.004) and a higher incidence of death or initiation of dialysis (adjusted HR, 1.48; 95% CI, 1.04 to 2.11; *p* = 0.03) (Figure 3).

Thus, in SCAD patients with advanced CKD, the invasive strategy did not prove beneficial and could be harmful.

## 5. Interim Results of ISCHEMIA-EXTEND

The primary endpoint rate crossover in the ISCHEMIA study, which occurred in the middle of the follow-up, with a statistically insignificant trend towards the advantage of the invasive strategy, urged the study leadership to organize an ISCHEMIA-EXTEND trial (NCT04894877) [23], resulting in an increase in the total follow-up duration to 7 years.

**Figure 4:**
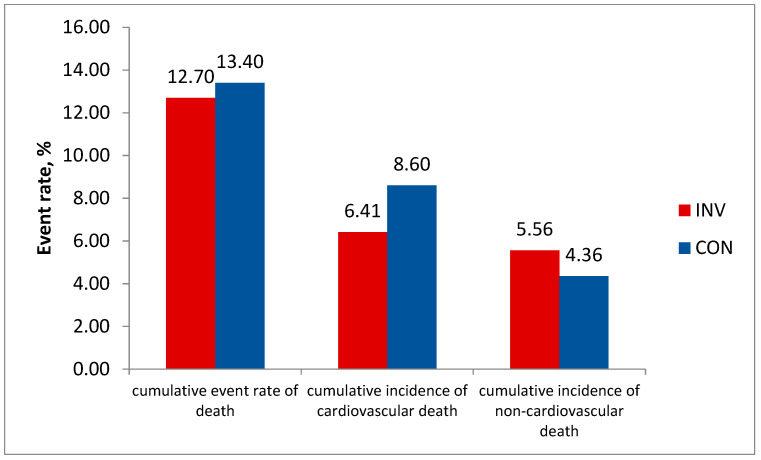
Total cardiovascular and non-cardiovascular mortality by treatment strategy in ISCHEMIA-EXTEND. *p* = NS for total mortality. *p* < 0.05 both for cardiovascular mortality and non-cardiovascular mortality.

The data published recently show lower long-term cardiovascular mortality at 7 years in the invasive vs. conservative group (6.41% vs. 8.60%, difference −2.19% (95% CI, −3.85% to −0.53%) and higher non-cardiovascular mortality (respectively, 5.56% vs. 4.36%, difference 1.20% (95% CI, −0.32% to 2.72%). This resulted in an equal total mortality rate in both groups: 12.70% vs. 13.40% (difference −0.70% (95% CI, −2.95% to 1.56%)) (Figure 4).

The authors also found an absence of significant interaction on the total mortality between the presence or absence of multivessel CAD and the randomized initial strategy. The cumulative all-cause mortality rate for participants with multivessel disease (MVD) was not lower with the invasive versus conservative management strategy: adjusted HR 0.89 (95% CI, 0.67 to1.19). However, the statistically significant benefit of an invasive strategy could be demonstrated for cardiovascular mortality in patients with MVD, adjusted HR 0.68 (95% CI, 0.48 to0.97), in contrast to the finding in the prespecified subgroup analysis of the main study. Meanwhile, non-cardiovascular mortality tended to be higher in MVD patients (adjusted HR 1.52 (95% CI, 0.88 to 2.63), both findings resembling those in the total ISCHEMIA EXTEND population). Unfortunately, no data were collected on nonfatal events after the initial median 3.2-year follow-up. Due to the trend toward better cardiovascular outcomes with the invasive strategy, predominantly in MVD, the ISCHEMIA-EXTEND group plans to continue to follow surviving participants into 2025 for a projected median of 10 years. As for the higher rate of noncardiovascular death in the invasive group, the authors note that it was unexpected and remains unexplained. The previous analysis showed more malignancy deaths (2.0% vs. 0.8%; HR 2.11 [1.23–3.60]) with the invasive strategy than with the conservative strategy [24], though the mechanism of this remains unclear and is not related to the radiation exposure of dual antiplatelet therapy [23].

### Myocardial Revascularization and Symptoms of Ischemia in SCAD

Despite the lack of a convincing effect on prognosis, the effect of treatment strategy on symptoms has been shown [17]. The subjective improvement in symptoms of patients after randomization was more pronounced in participants in the invasive strategy group, and the difference persisted throughout the entire follow-up period (thus, the average total SAQ scores compared with the conservative strategy group were 84.7 ± 16.0 vs. 81.8 ± 17.0 after 3 months; 87.2 ± 15.0 vs. 84.2 ± 16.0 after 12 months; and 88.6 ± 14.0 vs. 86.3 ± 16.0 after 36 months). The posterior mean difference in the SAQ Summary score favored the invasive strategy by 4.1 points at 3 months and by 4.2 points at 12 months. The magnitude of this benefit depended on angina frequency at baseline, with 35% of cases being asymptomatic. The greatest benefit was observed in the most symptomatic participants [17,25].

## 6. Discussion

### 6.1. ISCHEMIA Advantages

The problem of treatment strategy selection in SCAD remains one of the most controversial issues in clinical practice. The current role of revascularization has been extensively explored in the ISCHEMIA study, the largest RCT to date in patients with SCAD, and continued in the ISCHEMIA-EXTEND study. The ISCHEMIA design had a number of important advantages:

- Randomization occurred in the absence of information about the coronary angiography data, resulting in the inclusion of a significant number of patients with high-risk lesions;
- Moderately or strongly positive stress tests were the inclusion criterion. As a result, ISCHEMIA patients were quite “ill” as judged by the results of the stress test (in contrast to the COURAGE trial participants [26]), while in such patients the invasive strategy should theoretically have the greatest advantage over the conservative one [3];
- Only second-generation drug-eluting stents were used, and FFR was assessed in borderline lesions. As a result, revascularization of physiologically significant lesions was performed in most cases, similar to the FAME 2 study, which showed the prognostic advantage of an invasive strategy with this approach [11];
- As a method of revascularization, both PCI and CABG were used, the latter with mammary grafts as a rule (PCI only in COURAGE and FAME 2);
- For the diagnosis of unstable angina during follow-up, objective confirmation was required, the absence of which is considered the reason for the debatable results of the FAME 2 study [10];
- As a result, high-risk patients were included both in terms of functional and anatomical criteria, and invasive treatment was carried out at the optimal modern level. At the same time, even by the end of the follow-up, OMT was not ideal, with only 41% of patients receiving “true” OMT, and the target LDL-C value was more liberal than at present.

It was under these conditions that the important results of the study were obtained:

1. An invasive strategy, compared with a conservative strategy, did not result in a lower risk of the primary endpoint;
2. Low mortality was observed with both strategies, including the conservative strategy, in the population of patients with severe coronary artery disease. It was 5.5% per 3.2 years of follow-up, i.e., about 1.7%/year—which is about half as much as calculated for patients of this risk level on the stress test [7];
3. Invasive strategies showed no prognostic benefit in the prespecified important subgroups (strongly positive stress test; 3-vessel disease; proximal LAD disease). The advantage of an invasive strategy in the prevention of spontaneous AMI could be shown;
4. An advantage of an invasive strategy in reducing the symptoms of ischemia has been shown.

### 6.2. ISCHEMIA Limitations

ISCHEMIA has several limitations that have been widely discussed.

#### 6.2.1. Methodology and Enrollment

- Due to slow enrollment, the sample size had to be reduced from the originally planned 8,000 to approximately 5,000. Consequently, the primary endpoint was expanded from the cumulative of cardiovascular death or MI to a 5-component outcome to maintain statistical power [27]. However, the authors clearly indicated that the plan to adopt the 5-component primary endpoint if aggregate, blinded accruing data demonstrated a lower than expected event rate was finalized, approved by the Data Monitoring Board, and included in the original protocol version before any patients were enrolled in the trial [28];
- A high proportion of enrolled subjects in ISCHEMIA were asymptomatic or mildly symptomatic [25]. In our opinion, it could contribute to the higher exercise capacity at the stress test shown in ISCHEMIA (median 7.8 MET) [15] than it would be in a more symptomatic population. Such high exercise capacity probably does not reflect the typical profile of SCAD patients being referred to angiography and constitutes the study limitation;
- The patients inclusion was based on the stress test analysis performed locally to better reflect real clinical practice, which led to ≈15% having less than moderate ischemia according to the core laboratory review [19]. This fact resulted in somewhat exaggerated ischemia severity reported in the enrolled patients;
- As in patients with eGFR < 60 mL/min, CCTA was not performed, the coronary anatomy data was available only in 48% of the enrolled patients [19], which reduced the power of the related analyses;
- Patients with lesions of the LMCA and those with EF < 35%, as well as those with end-stage CKD, were not included in the study. At the same time, according to the analysis of the American Cardiovascular Data Registry (NCDR), among patients with SCAD undergoing PCI, 18.5% have a decreased EF, left main disease, or CKD stage 5 [29]. It should be noted that patients with LMCA have also been excluded from the key RCTs (COURAGE, BARI-2D, and FAME II) studying patients with SCAD, while the key trials comparing PCI and CABG in patients with LMCA (EXCEL, PRECOMBAT, and NOBLE) have shown conflicting results [6]. At the same time, the question of the appropriateness of revascularization to improve prognosis in patients with severe CKD was answered (negatively) in the ISCHEMIA-CKD sub-study;
- The requirements for the PCI procedure (intravascular imaging, plaque modification, implementation of FFR measurement, and completeness of revascularization) were not imposed by the protocol but left to the choice of the operator. This could have led to a non-universal approach to what was considered “revascularization” in the invasive arm and a heterogeneous ultimate revascularization quality;
- The crossover from the conservative arm to the invasive arm resulted in 21% of patients assigned to the conservative arm undergoing elective revascularization. Though this proportion is sizeable, the yearly crossover rate in ISCHEMIA was 6.6%, similar to the rate in COURAGE (7.2%) [16].

This said, ISCHEMIA was considered one of the most high-quality contemporary RCTs in cardiology [30].

#### 6.2.2. Results

The main limitation of the ISCHEMIA study is the “crossover” of the event curves for all major endpoints at 2 years after randomization, after which there was a trend towards an increase in the number of events in the conservative group. In addition, the analysis of outcomes in the most important “controversial” subgroups (item 3 above) was secondary, and the analysis of outcomes separately for types of revascularization (PCI and CABG) has not yet been published. Most of the separate PCI analyses previously failed to find its prognostic benefit over OMT in SCAD regarding the hard endpoints [31]. Meanwhile, while CABG has a prognostic advantage over PCI in SCAD [32], a contemporary randomized analysis of CABG vs. OMT is lacking.

### 6.3. ISCHEMIA Results Compared to Other New Research Data

The ISCHEMIA results are consistent with other new studies and meta-analyses. In particular, prolonged (15-year) follow-up of COURAGE participants showed no reduction in mortality after PCI in 3-vessel coronary artery disease—it was 50% and 53% for medical treatment and PCI, respectively [33]. Similarly, in patients with a high-risk stress test, at 15 years, these rates were 44% and 50%, respectively (all differences were non-significant).

In a large meta-analysis (14 RCTs with a total of 14,877 participants, mean follow-up 4.5 years), revascularization (PCI or CABG) did not reduce the risk of death compared to conservative therapy alone (HR 0.99 [95% CI 0.90 to1.09]) [34]. Revascularization reduced the risk of spontaneous MI (HR 0.76 [95% CI 0.67 to0.85]) but increased the incidence of periprocedural MI (HR 2.48 [95% CI 1.86 to3.31]). Finally, revascularization increased the likelihood of freedom from angina by 10% (HR 1.10 [95% CI 1.05 to1.15]).

An additional follow-up of ISCHEMIA participants (ISCHEMIA-EXTEND) up to 7 years confirmed the conclusion of the main study that there was no benefit of the invasive strategy in terms of overall mortality. This resulted from the opposite effects of a statistically significant decrease in cardiovascular mortality found in the invasive group (probably as a result of a decrease in the frequency of spontaneous AMI observed in the early period of follow-up) and a parallel unexpected increase in non-cardiac, apparently oncological, mortality. The significance of these data has yet to be assessed.

Table 2 compares the statements of the current European guidelines for myocardial revascularization with the data from ISCHEMIA.

Based on the results of ISCHEMIA, the American Cardiology Societies issued the new Clinical Guidelines [21], where only 3-vessel disease with reduced EF, as well as LMCA disease—i.e., subgroups of patients minimally or not represented in ISCHEMIA—are left as Class I/IIa indications for revascularization in SCAD.

At the same time, revascularization by CABG in patients with 3-vessel disease and preserved EF received a Class IIb recommendation. In this regard, the European Association for Cardio-Thoracic Surgery, the American Association for Thoracic Surgery, and the Society of Thoracic Surgeons issued statements disagreeing with this conclusion. As an argument, they indicate that this conclusion in the Guidelines is based on a subgroup analysis of the RCT, which itself is not randomized. Moreover, a separate analysis of the predictive value of revascularization depending on its type was not published at the time of publication of the Guidelines [35,36]. It could be added that the finding of lower cardiovascular mortality with the invasive strategy in patients with MVD in ISCHEMIA-EXTEND is an argument for the invasive strategy, though its effect on non-cardiovascular mortality in this patient group was not separately described.

## 7. Conclusions

The ISCHEMIA study generally demonstrated no prognostic benefit of a primary invasive strategy for individuals with SCAD and a moderate-to-high-risk stress test receiving current OMT on the cumulative cardiovascular endpoint. No advantage of revascularization regarding all-cause mortality was confirmed in the ISCHEMIA-EXTEND 7-year interim analysis. Also, revascularization was of no benefit in SCAD patients with advanced CKD. It can be concluded that a conservative strategy may be the primary choice for the majority of patients with SCAD who meet the ISCHEMIA eligibility criteria and do not have limiting symptoms. For other SCAD patients not meeting the ISCHEMIA eligibility criteria, the tailored strategy choice is advisable.

The ISCHEMIA findings conflicted with the current European guidelines, and given the size of the study and the high quality of the data, a revision of the recommendations seemed inevitable. However, the new ACC/AHA guidelines for revascularization based on the results of ISCHEMIA were not supported by the cardiac surgery communities regarding the appropriateness of surgical treatment of multivessel lesions. Indeed, conclusions about the insufficient prognostic effect of revascularization in patients with high-risk coronary anatomy in the main trial are based on secondary analysis. On the other hand, while the cardiovascular mortality benefit of invasive strategy in MVD patients was shown in the secondary analysis of ISCHEMIA EXTEND, it was offset by the unexpected rise of non-cardiovascular mortality. In this regard, the appropriateness of an invasive approach for patients with MVD should be specifically addressed and the conduct of an RCT with a direct comparison of invasive and conservative strategies in this category of patients looks justified and substantiated by the results obtained in ISCHEMIA and ISCHEMIA EXTEND. The same applies to patients with reduced EF and LMCA lesions, which were underrepresented or not represented in ISCHEMIA. These much-needed data will be able to complete the most up-to-date SCAD treatment strategy algorithm.

## Figures and Tables

**Table 1:** Class I and IIa indications for myocardial revascularization according to the ACC/AHA Guidelines [4].

Indication	Description
For prognosis	High risk coronary anatomy: unprotected LMCA disease three-vessel disease one- or two-vessel lesion involving the proximal LAD
	Two-vessel disease without proximal LAD involvement in the presence of large area of inducible ischemia
	Left ventricular EF reduction (35–50%)
	History of sudden cardiac death in the presence of documented ventricular tachycardia related to ischemia
For ischemic symptoms	≥1 coronary artery stenosis (or graft stenosis in a patient after CABG) and ‘unacceptable’ angina severity despite OMT or if OMT cannot be prescribed (contraindications, side effects, patient preferences)

LMCA—left main coronary artery; LAD—left anterior descending artery; LV EF—left ventricular ejection fraction; CABG—coronary artery bypass grafting; OMT—optimal medical therapy. Classes of recommendations are specified according to the usual definition. Angiographically significant lesions (≥50% for the left main coronary artery and ≥70% for other lesions) are implied throughout the text of the table. The prognostic benefit of revascularization is shown in most cases only for CABG; most clinical scenarios for revascularization to improve prognosis by percutaneous coronary intervention (PCI) are Class IIb recommendations.

**Table 2:** Indications for revascularization in patients with SCAD: European Guidelines versus ISCHEMIA data.

Current European Guidelines	New Research
**For prognosis**	
LMCA disease	?
Proximal LAD involvement	X
2- or 3-vessel disease	X
Large area of inducible ischemia (>10% of the left ventricle)	X
EF < 35%	?
**For ischemic symptoms**	
Hemodynamically significant coronary artery stenosis in the presence of limiting angina or angina equivalent, with insufficient response to OMT	✓

X—not confirmed; ?—not studied; ✓—confirmed.

## Data Availability

Publicly available datasets were analyzed in this study. The scope of this data is limited to the references list.
